# Effects of hyperbaric oxygen preconditioning on cardiac stress markers after simulated diving

**DOI:** 10.1002/phy2.169

**Published:** 2013-11-24

**Authors:** Arve Jørgensen, Philip P Foster, Alf O Brubakk, Ingrid Eftedal

**Affiliations:** 1 Department of Circulation and Medical Imaging, Norwegian University of Science and TechnologyTrondheim, Norway; 2 Department of Diagnostic Imaging, St. Olavs University HospitalTrondheim, Norway; 3 Division of Pulmonary, Sleep Medicine, and Critical Care, Departments of Internal Medicine and NanoMedicine and Biomedical Engineering, The University of Texas Health Science Center at HoustonTexas

**Keywords:** Cardioprotection, decompression illness, diving, gas embolism, hyperbaric oxygen preconditioning

## Abstract

Hyperbaric oxygen preconditioning (HBO-PC) can protect the heart from injury during subsequent ischemia. The presence of high loads of venous gas emboli (VGE) induced by a rapid ambient pressure reduction on ascent from diving may cause ischemia and acute heart failure. The aim of this study was to investigate the effect of diving-induced VGE formation on cardiac stress marker levels and the cardioprotective effect of HBO-PC. To induce high loads of VGE, 63 female Sprague–Dawley rats were subjected to a rapid ambient pressure reduction from a simulated saturation dive (50 min at 709 kPa) in a pressure chamber. VGE loads were measured for 60 min in anesthetized animals by the use of ultrasonography. The animals were divided into five groups. Three groups were exposed to either diving or to HBO-PC (100% oxygen, 38 min at 303 kPa) with a 45 or 180 min interval between HBO-PC and diving. Two additional groups were used as baseline controls for the measurements; one group was exposed to equal handling except for HBO-PC and diving, and the other group was completely unexposed. Diving caused high loads of VGE, as well as elevated levels of the cardiac stress markers, cardiac troponin T (cTnT), natriuretic peptide precursor B (*Nppb*), and *α*B-crystallin, in blood and cardiac tissue. There were strong positive correlations between VGE loads and stress marker levels after diving, and HBO-PC appeared to have a cardioprotective effect, as indicated by the lower levels of stress marker expression after diving-induced VGE formation.

## Introduction

The formation of gas emboli as a result of a reduction in ambient pressure (decompression) is a major cause of injury associated with diving (Vann et al. 2011). High loads of decompression-induced venous gas emboli (VGE) may result in cardiorespiratory decompression illness (DCI) with cough, dyspnea, pulmonary edema, shock and in the most severe cases, fatal outcome. Circulating VGE is effectively trapped in the lungs, and may cause increased pulmonary artery pressure, cardiac overload, and heart failure (Muth and Shank 2000). Moreover, blood perfusion and pulmonary gas exchange are impaired, and the arterial partial pressure of oxygen decreases relative to the increased number of VGE, resulting in hypoxia with subsequent cardiac ischemia and cell death (Butler and Hills 1985; Vik et al. 1990).

The phenomenon of preconditioning, in which a period of sublethal cardiac stress can protect the heart against injury during a subsequent ischemic insult, has been the subject of intense research over the last two decades (Yellon and Downey 2003). Hyperbaric oxygen (HBO), which has been used as a preconditioning stimulus prior to ischemia, has been shown to provide wide-scale cardioprotective effects (Cabigas et al. 2006; Yogaratnam et al. 2010). HBO preconditioning (HBO-PC) in rats exposed to simulated diving has recently shown promising results in reducing the incidence, severity, and complications of DCI (Martin and Thom 2002; Butler et al. 2006; Katsenelson et al. 2009; Fan et al. 2010; Ni et al. 2013). However, the potential cardioprotective effects of HBO-PC in relationship with gas emboli formation have not yet been investigated.

In this study, rats were exposed to a simulated dive followed by severe decompression stress inducing high loads of VGE. First, we aimed to investigate whether a simulated dive with subsequent VGE formation would lead to increased levels of cardiac stress markers indicating cardiac stress and injury, and second, investigate the effect of HBO-PC on these markers. Three different cardiac stress markers in rat serum and cardiac tissue were selected; serum cardiac troponin T (cTnT), a biomarker of cardiac injury (Thygesen et al. 2012); cardiac gene expression of the natriuretic peptide precursor B (*Nppb*), which is a biomarker of acute heart failure (Nakagawa et al. 1995; Braunwald 2008); and the cardiac gene and protein expression of *α*B-crystallin, a small heat shock protein with a key role in protecting the heart from injury (Latchman 2001; Whittaker et al. 2009; Christians et al. 2012). We hypothesized that high loads of VGE from simulated diving would result in an elevation of these cardiac stress markers (cTnT, *Nppb*, and *α*B-crystallin). We further hypothesized that HBO-PC would protect the heart from diving-induced VGE formation, resulting in lower levels of these stress markers.

## Material and Methods

### Ethical approval

The experimental protocols were approved by the Norwegian Committee for Animal Experiments, and were performed according to the Guide for the Care and Use of Laboratory Animals published by the Directive 2010/63/EU of the European Parliament.

### Experimental animals

A total of 63 adult female Sprague–Dawley rats (Taconic, Ry, Denmark), 285 ± 17 g (SD), were randomly assigned to five groups:

- Simulated diving, *n* = 12
- HBO-PC followed by a 45 min normobaric air interval between HBO-PC and simulated diving (HBO45), *n* = 12
- HBO-PC followed by a 180 min normobaric air interval between HBO-PC and simulated diving (HBO180), *n* = 12
- No diving, *n* = 18
- Unexposed, *n* = 9.

Groups I to IV were observed in anesthesia for 60 min after diving (gr. I–III) or no diving (gr. IV). Groups IV and V served as two different control groups. Group IV assessed the potential effect of anesthesia and handling without diving, and Group V was not exposed to anything (i.e., diving, chamber exposure, handling, or anesthesia). All the animals were housed in groups of three per cage in an animal facility. Light was controlled on a 12:12-h light-dark cycle at a room temperature of 21.0 ± 0.9°C (SD) and humidity 51 ± 9% (SD). The animals had free access to water and were placed on a pellet rodent diet.

### HBO preconditioning

Animals in Groups II (HBO45) and III (HBO180) were exposed to 100% oxygen for 5 min at normobaric pressure (101 kPa) in a pressure chamber, followed by an increase in ambient pressure (compression) at a rate of 200 kPa min^−1^–303 kPa. The animals were kept at that pressure for 38 min while breathing 100% oxygen. Because HBO exposure results in elimination of nitrogen gas (N_2_) from tissues (Foster and Butler 2009), the animals were exposed to air at the same ambient pressure (303 kPa) for 7 min immediately after the HBO exposure. According to the exponential model proposed by Foster et al. (1998), and using a critical tissue half-time (whole rat) of 10 min (Lillo and Parker 2000), this would cause N_2_ tissue tensions to differ ≤0.7 kPa between the groups prior to the dive. The rats were then decompressed at a rate of 200 kPa min^−1^ back to 101 kPa. The animals in the HBO45 and HBO180 groups were allowed to rest in their cages, breathing normobaric air, for 45 and 180 min, respectively, before simulated diving. The diving (gr. I) and nondiving (gr. IV) groups were exposed to normobaric air in a similar chamber at the same time, whereas the HBO45 and HBO180 animals were exposed to HBO.

### Simulated diving and VGE detection

The animals were compressed with air in a pressure chamber at a rate of 200 kPa min^−1^ from 101 to 709 kPa, breathing hyperbaric air for 50 min to obtain tissue saturation (Lillo and Parker 2000), and then decompressed linearly back to 101 kPa at a rate of 50 kPa min^−1^. Immediately after diving, the animals were anesthetized with a mixture of; midazolam 0.5 mg 100 g^−1^, fentanyl 5 *μ*g 100 g^−1^, and haloperidol 0.33 mg 100 g^−1^, which was administered as one bolus subcutaneous injection. The pulmonary artery and ascending aorta were insonated for 60 min using a 10 MHz transducer connected to a GE Vingmed Vivid 5 scanner. Gas emboli appeared in the pulmonary artery and aorta as bright spots and were recorded for 1 min at discrete time points (15, 30, and 60 min). The data were stored and played back in slow motion for analysis, in which the images were then graded (scan grade 0–5) according to a previously described method by an observer blinded to the experimental condition of the rats (Eftedal and Brubakk 1997). Scan grades were converted to the number of emboli·cm^2^·heart cycle^−1^ as previously described by Nishi et al. (2003). Animals that did not survive the 60 min postdive observation period due to severe DCI were excluded from further analysis. Animals in Groups I–IV were handled equally except for the differences in pressure profiles and breathing gas compositions.

### Serum cTnT analysis

After the 60 min postdive observation period, the abdomen was opened and blood from the abdominal aorta was collected into serum tubes. The serum used for the cTnT measurements was prepared by centrifugation at 10,000 rpm at 4°C after blood collection. A high-sensitivity cTnT assay (hs-cTnT; Roche Modular System E, Roche Diagnostics GmbH, Mannheim, Germany) was used to detect an elevation in cTnT (Giannitsis et al. 2010). This assay permitted the measurement of concentrations ≥10 ng L^−1^ (Omland et al. 2009).

### Preparation of myocardial tissue for mRNA and protein analysis

Immediately after blood sampling, the thoracic cavity was opened and approximately 50 mg of myocardial tissue sections of the right and left ventricle were rapidly excised and rinsed in RNAlater buffer solution (Ambion Inc., Austin, TX). The tissue was then transferred to 1.5 mL fresh RNAlater and kept at room temperature for up to 4 h before storage at −80°C. To prepare the lysates for mRNA and protein analysis, the myocardial tissue was thawed at room temperature, weighed and then transferred into 5 mL round-bottom polystyrene tubes containing 10 volumes per tissue weight (*μ*L mg^−1^) of RNeasy Fibrous Tissue lysis buffer (Qiagen, Valencia, CA) and was mechanically disrupted using an UltraTurrax rotor/stator (IKA Werke GmbH & Co., Staufen, Germany) until completely homogenized. The lysate was split into two equal volumes: one part was used for real-time reverse transcription polymerase chain reaction (qRT-PCR) analysis, where the total RNA was extracted on a Qiacube nucleic acid extractor using the RNeasy Fibrous Tissue mini kit (Qiagen) according to the manufacturer's recommendations; and the other part was used for western blotting analysis, where 1% protease inhibitor solution (Qiagen) was added into the lysate, and the sample was precipitated by adding an equal volume of 10% ice-cold TCA followed by incubation on ice for 20 min. After centrifugation (16000 *g*), the protein pellet was washed with 100% ethanol and then resuspended in 150 *μ*L loading buffer (Invitrogen, Carlsbad, CA).

### *Nppb* and αB-crystallin gene expression

Prior to the analysis, the total RNA concentration and purity was determined using a NanoDrop 2000 spectrophotometer (NanoDrop Technologies, Wilmington, DE) (Schroeder et al. 2006). The mRNA expression levels of the genes encoding for the natriuretic peptide precursor B (*Nppb*) as well as the small heat-shock protein, *α*B-crystallin, were analyzed in the left and right cardiac ventricles for each of the five treatment groups (*n* = 6–8 from each group by random selection) with qRT-PCR using Qiagen QuantiFast FAM-labeled target probe assays with the QuantiFast Probe RT-PCR Plus kit (Qiagen) in a one-step qRT-PCR normalized against MAX-labeled *Hprt*. The PCR was run on a C1000 thermal cycler (Bio-Rad, Pleasanton, CA) with a CFX96 Optical Reaction Module and analyzed on the CFX Manager Software version 2.0 using the ΔΔC_T_ method in which the relative quantity of the target genes was normalized against the relative quantity of the control across the samples (Livak and Schmittgen 2001).

### αB-crystallin protein expression

Resuspended protein lysates from each of the five treatment groups (*n* = 5 from each group by random selection) were analyzed using 1D polyacrylamide gel electrophoresis in 10% NuPage Novex Bis-Tris gels (Invitrogen) in one (3-(N-morpholino) propanesulfonic acid) electrophoresis buffer. The gels were run at the same time before they were electroblotted onto nitrocellulose membranes and blocked for 1 h in 5% fat-free dry milk diluted in phosphate-buffered saline (PBS) + 0.1% Tween (PBST). The membranes were first incubated together with primary antibody against *α*B-crystallin (ADI-SPA-222; Enzo Life Sciences, Farmingdale, NY) for 1 h in blocking buffer, washed for 3 × 10 min in PBST, further incubated for 1 h in a secondary IRDye-conjugated antibody in PBST and finally washed 3 × 10 min in PBST and 1 × 10 min in PBS. As a loading control, the membranes were then treated with an antibody against *β*-tubulin (AB6046; AbCam, Cambridge, MA). All membranes were probed with the same batch of *β*-tubulin control to ensure that the protein quantification was equally performed across all samples and gels. The fluorescence signals were detected using an Odyssey scanner (Li-Cor Biosciences, Lincoln, NE) and the signal intensities and relative protein quantification were calculated using Image Studio 2.0 (Li-Cor Biosciences).

### Statistical analysis

The data were expressed as the median with ranges or as the mean ± SEM. We employed nonparametric tests due to the limited number of rats. The Mann–Whitney *U*-test and Kruskal–Wallis test were used to evaluate the differences in VGE loads and the cardiac stress markers, cTnT, *Nppb*, and *α*B-crystallin, between the groups. Fisher's exact test was used to evaluate the ratio of animals in each group, and the ratio of animals with low (grades 0–3) or high (grades 4–5) bubble grades, that had cTnT values above the detection limit. Selected bivariate relationships were examined using Spearman's rank correlation test. *P* < 0.05 was considered statistically significant. On the basis of the estimates obtained from previous studies (Wisloff and Brubakk 2001; Wisloff et al. 2004), 12 rats in each of the three diving groups would provide a power of 0.86.

## Results

### VGE loads detected by ultrasonography

**Figure 1:**
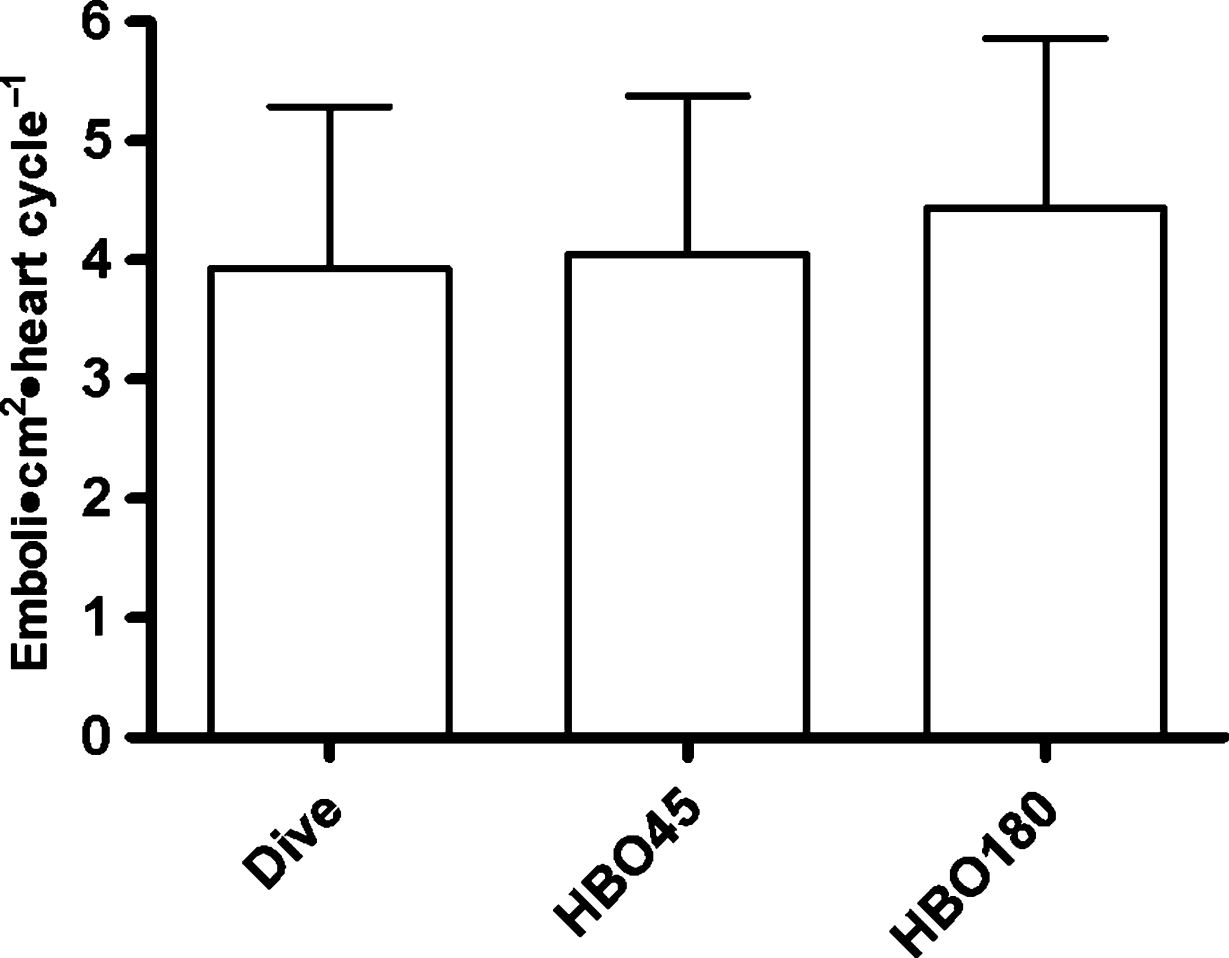
The maximum number of venous gas emboli (VGE) in the pulmonary artery after diving, which was measured as emboli·cm^2^·heart cycle^−1^, did not differ between the three groups of diving rats (*n* = 12 in each group). HBO45/HBO180: hyperbaric oxygen preconditioning (HBO-PC) followed by a 45 or 180 min rest interval between HBO-PC and diving. The data are presented as the means ± SEM.

The diving protocol resulted in a 25% mortality rate in all the animal groups during the first hour after diving. The animals that died had massive amounts of VGE with a scan grade 5 (on a scale from 0 to 5) or ∼10 emboli·cm^2^·heart cycle^−1^. HBO-PC had no effect on VGE formation, which was measured as the maximum amount of emboli·cm^2^·heart cycle^−1^ (Dive: 3.9, HBO45: 4.1, HBO180: 4.4, *P* = 0.92, *n* = 12 in each group, Fig. 1). No gas emboli were detected in the ascending aorta of any of the animals that survived the observation period.

### Serum cTnT levels

**Figure 2:**
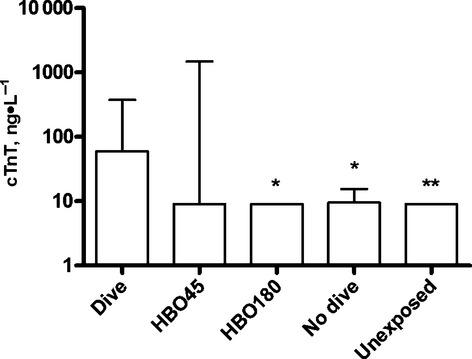
Hyperbaric oxygen preconditioning resulted in reduced postdiving serum cardiac troponin T levels. Serum cardiac troponin T (cTnT) levels (ng L^−1^) were higher in the diving (*n* = 10) compared to the HBO180 (*n* = 7), nondiving (*n* = 18) and unexposed (*n* = 9) animals, but were not different from the HBO45 (*n* = 9) animals. The cTnT level in the animals with values below the detection limit (10 ng L^−1^) was established at 9 ng L^−1^. The data are presented as the median ± interquartile range. All the animals were handled equally and differed only in the pressure and breathing gas exposures, except for the unexposed animals, which were kept shielded in their cages until further blood sampling. HBO45/HBO180: hyperbaric oxygen preconditioning (HBO-PC) followed by a 45 or 180 min rest interval between HBO-PC and diving. *P < 0.05, **P < 0.001 significantly different from the diving animals.

Animals exposed to simulated diving (gr. I) demonstrated significantly higher levels of serum cTnT compared to the nondiving (gr. IV, *P* = 0.02), unexposed (gr. V, *P* = 0.0007) and HBO180 (gr. III, *P* = 0.01) animals, but were not different from the HBO45 (gr. II) animals (Fig. 2). A significantly higher percentage (80%) of non–preconditioned diving animals (gr. I) showed elevated cTnT levels (above the detection limit) compared to the preconditioned HBO180 (13%, *P* = 0.02) and unexposed animals (0%, *P* = 0.0007). There was a positive correlation between the amount of cTnT and VGE in all the diving animals (*r*_s_ = 0.66, *P* = 0.0002).

Preconditioned animals appeared to tolerate higher VGE loads compared to non–preconditioned animals. For example, in animals with a scan grade of less than 4 (*n* = 18), elevated cTnT levels were found in only 1/12 of the preconditioned animals in contrast to 4/6 of the non–preconditioned animals (*P* = 0.02). All the animals with a scan grade ≥4 (*n* = 9) demonstrated elevated levels of cTnT.

### Cardiac *Nppb* gene expression

**Figure 3:**
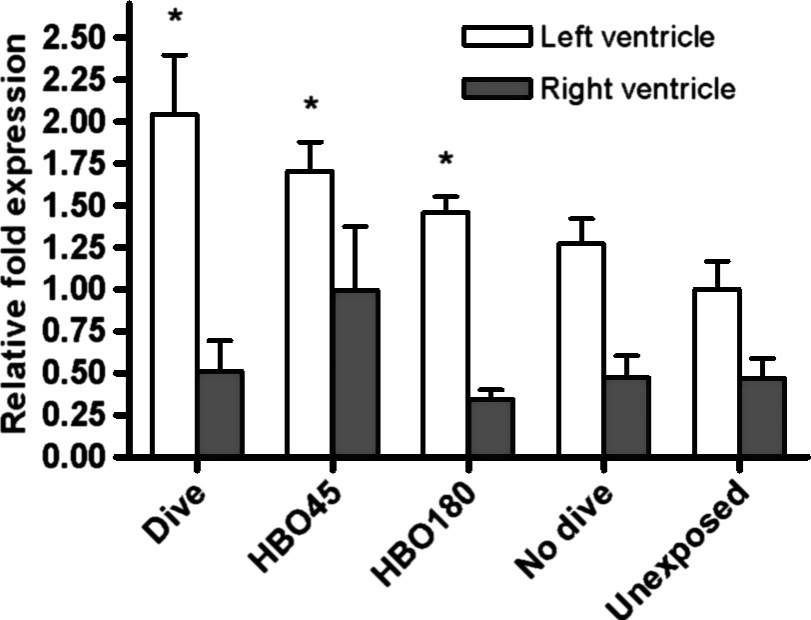
Cardiac tissue level of brain natriuretic peptide precursor (*Nppb*) was increased after simulated diving. *Nppb* mRNA expression was increased in the left cardiac ventricle in all the diving animal groups compared to unexposed animals. Differences were shown as the relative fold expression compared to the control gene *Hprt*. All the animals were handled equally and differed only in the pressure and breathing gas exposures, except for the unexposed animals, which were kept shielded in their cages until further tissue sampling. HBO45/HBO180: hyperbaric oxygen preconditioning (HBO-PC) followed by a 45 or 180 min rest interval between HBO-PC and diving. Values were expressed as the means ± SEM, *n* = 6–8 in all groups. *P < 0.05 significantly different from the unexposed animals.

The diving animals (gr. I), HBO45 (gr. II), and HBO180 (gr. III) showed a 2.0-fold (*P* = 0.02), 1.7-fold (*P* = 0.01), and 1.5-fold (*P* = 0.03) increase in *Nppb* expression in the left ventricle compared to the unexposed animals (gr. V), respectively (Fig. 3). No differences in *Nppb* expression were observed between the nondiving (gr. IV) and unexposed animals. *Nppb* expression levels were positively correlated with cTnT (left ventricle: *r*_s_ = 0.65, *P* = 0.00001, right ventricle: *r*_s_ = 0.38, *P* = 0.02) and VGE (left ventricle: *r*_s_ = 0.44, *P* = 0.04, right ventricle: *r*_s_ = 0.47, *P* = 0.02).

### αB-crystallin gene and protein expression

**Figure 4:**
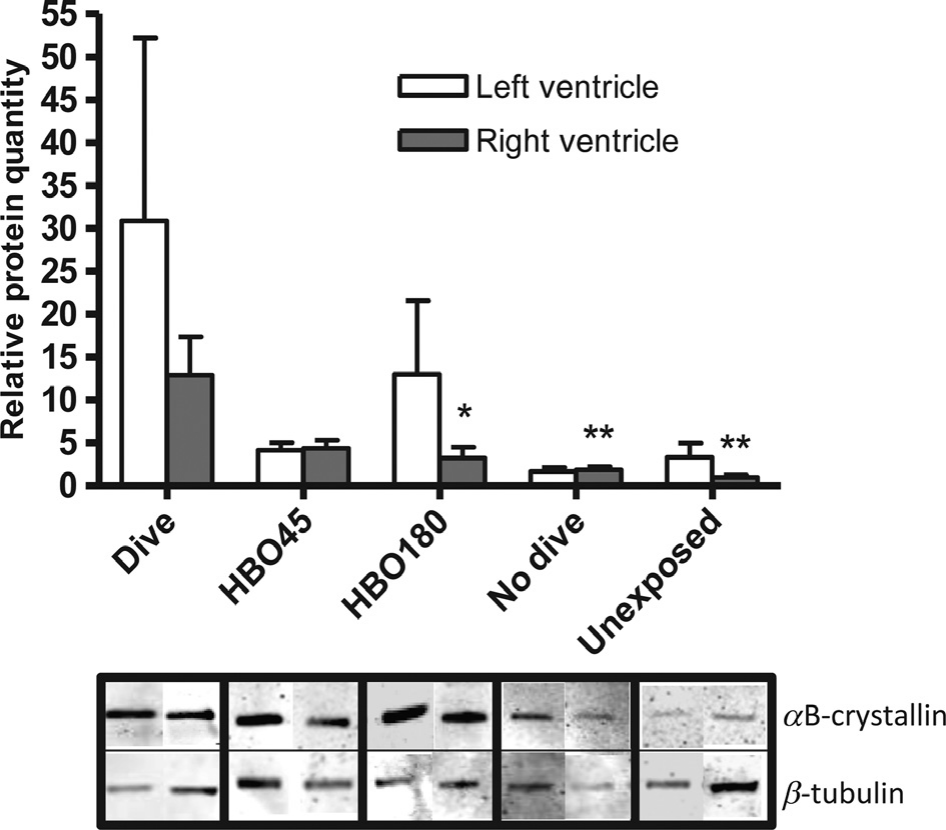
Hyperbaric oxygen preconditioning (HBO-PC) resulted in reduced postdiving cardiac *α*B-crystallin protein levels. Relative protein levels of *α*B-crystallin in the right cardiac ventricle of diving animals were increased compared to HBO180, nondiving and unexposed animals. The differences were shown as the relative protein quantity compared to the *β*-tubulin control. For each group, a representative western blot from one animal is shown. All the animals were handled equally and differed only in the pressure and breathing gas exposures, except for the unexposed animals, which were kept shielded in their cages until further tissue sampling. HBO45/HBO180: HBO-PC followed by a 45 or 180 min rest interval between HBO-PC and diving. Values were expressed as the means ± SEM, *n* = 5 in all groups. *P < 0.05, **P < 0.01 significantly different from the diving group.

None of the groups showed altered levels of *α*B-crystallin mRNA expression in cardiac tissue after simulated diving. However, the relative protein level of *α*B-crystallin in the non–preconditioned diving animals (gr. I) was increased by 4.0-fold, 6.9-fold, and 12.6-fold in the right ventricle compared to the HBO180 preconditioning (gr. III, *P* = 0.02), nondiving (gr. IV, *P* < 0.01) and unexposed animals (gr. V, *P* < 0.01, Fig. 4). In addition, *α*B-crystallin in the right ventricle was positively correlated with cTnT (*r*_s_ = 0.72, *P* = 0.00005).

## Discussion

The primary findings of this study were that strenuous simulated diving with subsequent VGE formation resulted in increased levels of cardiac stress marker expression (cTnT, *Nppb*, and *α*B-crystallin) in rat serum and cardiac tissue. Moreover, HBO-PC prior to the dive appeared to provide cardioprotection, as indicated by the lower expression levels of these stress markers. The HBO-PC effect was more pronounced when there was a longer (180 min compared to 45 min) interval between HBO-PC and diving. In addition, a strong positive correlation was found between the amount of VGE and stress marker levels in the serum and cardiac tissue.

Elevated serum levels of cTnT induced by simulated diving were positively correlated with VGE loads. Cardiac troponins are components of the contractile apparatus in cardiomyocytes and demonstrate nearly absolute cardiac tissue specificity and high clinical sensitivity (Omland et al. 2009; Thygesen et al. 2012), and are the preferred biomarkers for the diagnosis of cardiac injury. Thus, our findings indicated that VGE formation after diving induced cardiac injury. The diving protocol resulted in a 25% mortality rate due to the massive amounts of VGE (scan grade 5), and all the surviving animals with a scan grade ≥4 showed elevated cTnT levels. Experiments by Butler and Hills (1985) and Vik et al. (1990) demonstrated a proportional relationship between VGE loads and impeded gas exchange with systemic hypoxia and cardiac overload. These previous findings and the increase in cardiac stress markers in this study, indicate that the diving protocol induced severe decompression stress with cardiorespiratory DCI. However, in the rats that died, there may of course have been injuries to other organ systems (e.g., brain and spinal cord) that contributed to the cause of death. However, in surviving rats no gas emboli were detected in the systemic circulation; injuries to other organ systems than the cardiopulmonary are therefore less likely. Cardiorespiratory manifestations of DCI are rare and have been reported to occur in approximately 2–6% of recreational diving accidents (Francis and Mitchell 2003a; Vann et al. 2011). Such manifestations only occur after highly provocative dives and represent a lethal form of DCI. If not treated immediately, acute heart failure may progress into cardiorespiratory collapse and death (Francis and Mitchell 2003a). Currently, there have been no published controlled diving studies of troponin release in animals or humans, but elevated troponins due to diving has been previously described in a case report (Chenaitia et al. 2010). Thus, this is the first study to demonstrate an association between gas emboli formation and troponin release after diving.

HBO-PC 180 min prior to simulated diving resulted in lower cTnT levels compared to non–preconditioned diving animals (Fig. 2) despite no differences observed in the VGE loads (Fig. 1). Thus, it appears that HBO-PC protects the heart against injury from decompression-induced VGE. This novel observation was consistent with the findings obtained by Martin and Thom (2002) and Butler et al. (2006), who demonstrated that similar HBO-PC protocols prior to simulated diving protected rats against severe decompression stress without reducing gas emboli formation. Martin and Thom found that HBO-PC reduced DCI manifestations from the central nervous system, and Butler et al. (2006) showed that HBO-PC resulted in less overall signs of DCI compared to control animals, and demonstrated lower levels of inflammatory markers in the blood, lungs, and urine after the dive.

In this study, two of the animals in the HBO45 group exhibited very high VGE loads (scan grade 4 and 5) throughout the entire 60 min postdiving observation period. These two rats had the highest cTnT levels measured, which may explain why the HBO45 group did not result in statistically significant lower levels of serum cTnT compared to the diving group. In the animals with low-to-moderate VGE loads (scan grade 0–3), significantly more animals showed elevated cTnT levels in the non–preconditioned group (67%) compared to the preconditioned groups (8%). However, all the animals with high VGE loads (scan grade 4 or 5) exhibited elevated cTnT levels. Thus, HBO exposure appeared to protect the heart against low-to-moderate loads of VGE; however, this protective effect was not evident when the VGE loads were high.

*Nppb* expression in the left ventricle was increased in all the diving groups compared to control animals (Fig. 3), and this increase was associated with increased cTnT and VGE levels. *Nppb* mRNA encodes for BNP and is a well-established biomarker of acute heart failure (Braunwald 2008). This natriuretic peptide is synthesized and released by cardiomyocytes in response to hemodynamic stress when the ventricles are subject to increased wall tension. The action of this peptide functions to oppose the physiological abnormalities that occur during cardiac overload and acute heart failure. It is shown that the expression of *Nppb* is increased within 1 h in response to cardiac overload (Nakagawa et al. 1995). Thus, the increase in *Nppb* expression in the left cardiac wall after simulated diving in this study indicated that the heart was exposed to increased wall tension and cardiac overload. During and after decompression, VGE is thought to form or grow on the endothelial surface in peripheral tissues until they are swept away by the bloodstream and trapped in the small vessels of the pulmonary circulation (Stepanek and Webb 2008). In animal studies, VGE trapped in the lungs caused increased pulmonary artery pressure, which may result in cardiac overload and heart failure (Bove et al. 1974; Vik et al. 1990). Although, this phenomenon has not been shown in human studies (Valic et al. 2005), observations after highly provocative dives with severe cardiorespiratory DCI indicated that heart failure and cardiac arrest may occur due to a massive VGE load in the pulmonary vascular bed (Muth and Shank 2000; Francis and Mitchell 2003b).

The elevation of cardiac stress markers in this study reflected VGE-induced cardiac stress and injury but provided no indication of the mechanisms behind this elevation. However, it is well known that cardiac ischemia, pulmonary embolism, and acute heart failure may all result in elevated cTnT and BNP levels (Giannitsis et al. 2000, 2010; Thygesen et al. 2012). Additional measurements of cardiopulmonary hemodynamics (e.g., pulmonary artery pressure and arterial oxygenation) in this study, could have added further information about the mechanisms.

The concept of cardiac preconditioning has been extensively studied (Hausenloy and Yellon 2011), and HBO-PC is one strategy used to induce cardiac protection. Exposure to HBO is thought to induce a cardiac stress response of a reduced intensity that initiates cardioprotective responses, thereby reducing the damage caused by subsequent, more severe stress. The functional basis for the protective effects of HBO preconditioning is only partially understood. Protective mechanisms of HBO involve increased oxidative stress, which induces heat-shock proteins, nitric oxide production, antioxidant enzymes, and modulates inflammatory responses (Nishizawa et al. 1999; Martin and Thom 2002; Cabigas et al. 2006; Thom 2009; Christians et al. 2012). In this study we investigated the effect of HBO preconditioning on the small heat-shock protein *α*B-crystallin, which interacts with cardiac proteins and is likely to play a key role in protecting the heart from cardiac overload and ischemia (Martin et al. 1997; Latchman 2001; Kumarapeli et al. 2008; Christians et al. 2012). *α*B-crystallin can protect the heart against various stresses by binding to and stabilizing cytoskeletal structures. In addition, *α*B-crystallin exhibits antiapoptotic and immunomodulatory properties, and administration of *α*B-crystallin is likely to diminish the extent and severity of ischemic lesions, including cardiac infarction, stroke, and arterial occlusion (Ousman et al. 2007; Arac et al. 2011). We found that *α*B-crystallin protein levels were increased in cardiac tissue after diving, and that HBO-PC rats displayed lower *α*B-crystallin levels compared to non–preconditioned rats. Thus, the high *α*B-crystallin levels in the heart after diving, may reflect that the heart have been exposed to a high level of diving-induced stress. Furthermore, the significantly lower *α*B-crystallin levels after HBO-PC and diving may reflect that HBO-PC induced a mild stress to the heart activating the cardioprotective properties of *α*B-crystallin. The activation of *α*B-crystallin prior to the dive could probably have prevented a subsequent higher increase in *α*B-crystallin levels after diving, because the heart was already preconditioned against the stress from diving. However, *α*B-crystallin mRNA expression levels were not significantly affected by HBO-PC or simulated diving. Therefore, the increased levels of *α*B-crystallin in response to diving are most likely initiated by protein stabilization and/or activation rather than by de novo transcription. On the basis of previous and present findings, it is likely that *α*B-crystallin plays a central role in protecting the heart against the injury from diving-induced VGE.

A limitation of this study was that the *α*B-crystallin protein samples from different groups were run on different membranes, and caution should therefore be used when interpreting this data.

In this study, cardiac stress markers were positively correlated with VGE. However, a recent study has shown that even presumably safe dives with few VGE and no DCI symptoms were associated with significant cardiac strain and increased levels of BNP (Marinovic et al. 2010). Grassi et al. (2009) showed that a water dive that was considered safe resulted in increased plasma BNP levels and that the same dive simulated in a dry hyperbaric chamber did not affect BNP levels. While underwater, divers are exposed to immersion resulting in significant hemodynamic changes (Pendergast and Lundgren 2009). When investigating how decompression-induced VGE formation affects the cardiovascular system, factors known to affect hemodynamics must be controlled. Simulated diving in a dry pressure chamber, such as the one used in this study, will eliminate the hemodynamic effects of immersion, enabling the differentiation between the effects of immersion and VGE on the cardiovascular system. However, a limitation of chamber diving is that dry diving is not the same as water diving with regards to cardiorespiratory stress.

HBO exposure is the main treatment of gas embolism and DCI after diving (Vann et al. 2011); however, vascular gas embolization can also result from a reduction in ambient pressure in caisson work, aviation, extravehicular activity during spaceflight or escape from pressurized vessels (Gennser and Blogg 2008; Vann et al. 2011), as well as from gas entry into the vasculature during inhospital procedures, for example, cardiac surgery with extracorporeal bypass and through central venous and hemodialysis catheters (Tibbles and Edelsberg 1996; Muth and Shank 2000). Thus, the implication of this study is that HBO may not only be used in the treatment of DCI after diving but also in a prophylactic manner to prevent DCI and/or injury due to vascular gas embolization during inhospital procedures. However, whether these novel findings can be translated into humans requires further investigation. A better understanding of HBO-PC mechanisms is important because it will facilitate preventive measures that will increase the safety of persons in risk of vascular gas embolization.

In conclusion, we found that the cardiac stress markers, cTnT, *Nppb,* and *α*B-crystallin, are elevated in rat serum and cardiac tissue after inducing high loads of VGE from simulated diving, and that there is a strong positive correlation between stress marker levels and postdive VGE loads. We have further shown that HBO-PC may prevent cardiac injury induced by gas embolism, as indicated by the reduced levels of these cardiac stress markers.
